# ICTV Virus Taxonomy Profile: *Ampullaviridae*

**DOI:** 10.1099/jgv.0.001023

**Published:** 2018-02-08

**Authors:** David Prangishvili, Mart Krupovic, ICTV Report Consortium

**Affiliations:** Department of Microbiology, Institut Pasteur, 25 rue du Dr Roux, 75015 Paris, France

**Keywords:** *Ampullaviridae*, ICTV Report, Taxonomy, Acidianus bottle-shaped virus

## Abstract

The family *Ampullaviridae* includes viruses with linear dsDNA genomes that replicate in hyperthermophilic archaea from the genus *Acidianus*. The virions have a unique champagne bottle-shaped morphology and consist of a nucleoprotein filament condensed into a cone-shaped core, which is encased by an envelope, with the base of the ‘bottle’ decorated with a ring of 20 filaments. Genome replication is presumably carried out by the virus-encoded protein-primed family B DNA polymerase. The bottle-shaped morphology is unprecedented among viruses of bacteria and eukaryotes and represents a group of archaea-specific virion morphotypes. This is a summary of the International Committee on Taxonomy of Viruses (ICTV) Report on the taxonomy of the *Ampullaviridae*, which is available at www.ictv.global/report/ampullaviridae.

## Virion

**Fig. 1.**
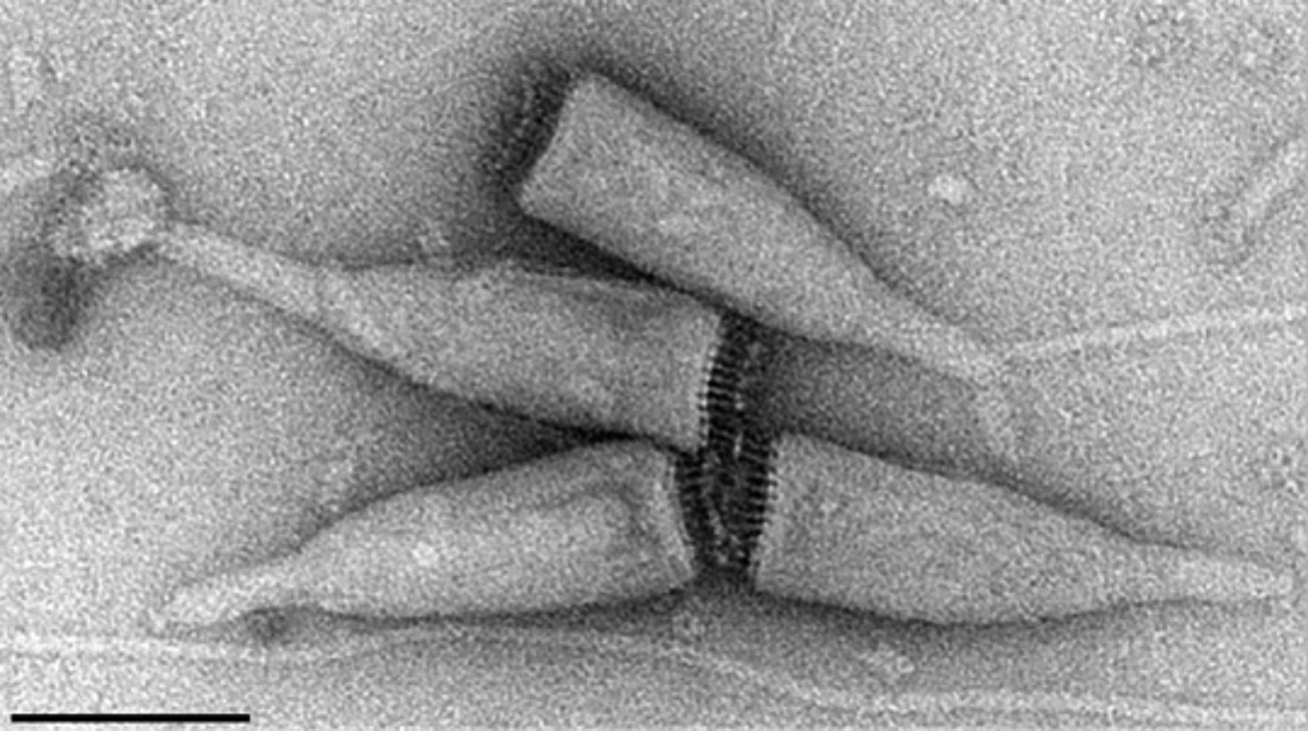
Negative-contrast electron micrographs of virions of an isolate of Acidianus bottle-shaped virus. The scale bar represents 100 nm. (Modified with permission from [1]).

The distinctive bottle-shaped virions are 230±20 nm long, and vary in width from 75 nm at the broad end, tapering to 4±1 nm. The twenty thin filaments at the broad end are each 20×3 nm, regularly spaced and interconnected via a basal disc or ring (Table 1, Fig. 1) [1].

**Table 1.** Characteristics of the family *Ampullaviridae*

Typical member:	Acidianus bottle-shaped virus (EF432053), species *Acidianus bottle-shaped virus*, genus *Ampullavirus*
Virion	Bottle shaped; 230 nm long, 4–75 nm wide; the flat terminus is decorated with 20 nm-long filaments; the envelope encases a cone-shaped nucleoprotein core
Genome	Linear, dsDNA (23 814 bp) with 590 bp terminal inverted repeats
Replication	Virus-encoded protein-primed family B DNA polymerase
Translation	Not characterized
Host range	Hyperthermophilic archaea from the genus *Acidianus*; non-lytic
Taxonomy	Single genus with a single species; two related genomes have been obtained from metagenomics studies

A 9 nm-thick virion envelope encases a cone-shaped core formed by a torroidally supercoiled nucleoprotein filament, which is 7 nm in width.

## Genome

**Fig. 2.**
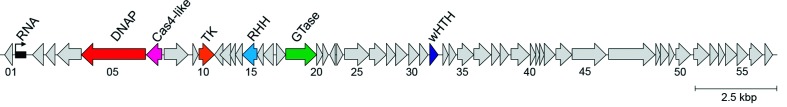
Genome organization of Acidianus bottle-shaped virus, showing the location, size and direction of putative genes. The black square indicates the position of a putative non-coding RNA gene. Functionally annotated genes are highlighted with different colours. Numbers below the genome diagram are ORF identifiers. Abbreviations: DNAP, DNA polymerase; Cas4-like, Cas4-like nuclease; TK, thymidylate kinase; RHH, ribbon*–*helix*–*helix motif; GTase, glycosyltransferase; wHTH, winged helix*–*turn*–*helix motif.

The genome is a linear double-stranded DNA molecule of 23 814 bp with 590 bp inverted terminal repeats. It has a base composition of 35 % GC and is predicted to encode 57 proteins [2] (Fig. 2). Three genes contain putative internal start codons with ribosome-binding sites. The genome encodes a DNA polymerase, a putative glycosyltransferase, a thymidylate kinase, a Cas4-like endonuclease and two putative DNA-binding proteins with winged helix*–*turn*–*helix and ribbon*–*helix*–*helix motifs, respectively. All of these proteins are conserved in two other ampullavirus genomes described from metagenomics studies [3]. The other predicted proteins have no known homologues. The genome also encodes a putative non-coding RNA, hypothesized to be involved in genome packaging [2].

## Replication

The viral DNA polymerase is homologous to protein-primed family B DNA polymerases and is apparently responsible for genome replication [2]. Virus adsorption appears to occur through the pointed end of the virion [1]. The virions are released without apparent host cell lysis.

## Taxonomy

The single genus *Ampullavirus* includes the single species *Acidianus bottle-shaped virus*. Related, unclassified, viruses have been identified by metagenomics studies of material from hot springs in Iceland, Italy and the USA [3]. Protein-primed DNA polymerases homologous to that encoded by Acidianus bottle-shaped virus have also been described in members of the archaeal virus genera *Gammapleolipovirus* (family *Pleolipoviridae*) and *Salterprovirus*, as well as in bacterial and eukaryotic viruses of the families *Tectiviridae*, *Podoviridae* (subfamily *Picovirinae*), *Adenoviridae* and *Lavidaviridae* (genus *Mavirus*) [4–6].

## Resources

Full ICTV Online (10th) Report: www.ictv.global/report/ampullaviridae.
